# Human Responses in Public Health Emergencies for Infectious Disease Control: An Overview of Controlled Topologies for Biomedical Applications

**DOI:** 10.1155/2022/6324462

**Published:** 2022-09-01

**Authors:** Kamal Naseri, Hasan Aliashrafzadeh, Maryam Otadi, Farnoosh Ebrahimzadeh, Homayoun Badfar, Iraj Alipourfard

**Affiliations:** ^1^Department of Architecture and Urban Studies (DAStU), Politecnico di Milano, Milan, Italy; ^2^Department of Medicine, Tabriz University of Medical Sciences, Tabriz, Iran; ^3^Chemical Engineering Department, Central Tehran Branch, Islamic Azad University, Tehran, Iran; ^4^Department of Internal Medicine, Faculty of Medicine, Mashhad University of Medical Sciences, Mashhad, Iran; ^5^Department of Mechanical Engineering, Urmia University of Technology (UUT), PO Box: 57166-419, Urmia, Iran; ^6^Institute of Biology,Biotechnology and Environmental Protection, Faculty of Natural Sciences, The University of Silesia in Katowice, Katowice, Poland

## Abstract

COVID-19 originated in Wuhan city of Hubei Province in China in December three years ago. Since then, it has spread to more than 210 countries and territories. This disease is caused by Severe Acute Respiratory Syndrome Coronavirus 2. The virus has a size of one to two nanometers and a single-stranded positive RNA. Droplets spread the virus from coughing and sneezing. This condition causes coughing, fever, acute respiratory problems, and even death. According to the WHO, the virus can survive outside the body for several hours. This research aimed to determine how environmental factors influenced the COVID-19 virus's survival and behavior, as well as its transmission, in a complex environment. Based on the results, virus transmissions are influenced by various human and environmental factors such as population distribution, travel, social behavior, and climate change. Environmental factors have not been adequately examined concerning the transmission of this epidemic. Thus, it is necessary to examine various aspects of prevention and control of this disease, including its effects on climate and other environmental factors.

## 1. Introduction

**Figure 1:**
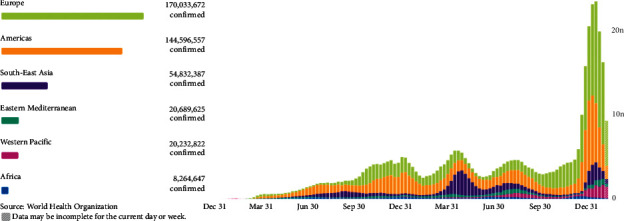
Global weekly incident cases of COVID-19 by World Health Organization Region as of February 15, 2022.

In late 2019, “coronavirus (COVID-19)” appeared in Wuhan, China [1], and was reported by the WHO as a new cause of pneumonia [2]. Based on the genomic sequence, the virus belongs to the genus Beta-coronavirus [3], which leads to hard illness and even death [4]; on the other hand, alpha-coronaviruses lead to mild or asymptomatic infections [5]. The possible route of transmission of COVID-19 infection is through person-to-person contact with contaminated surfaces by droplets that spread when coughing or sneezing [6]. The virus enters host cells by binding to the angiotensin-converting enzyme (ACE2) receptor [7]. The average period of incubation is 5.2 days [8]. People with underlying diseases like chronic cardiovascular disease, diabetes, lung disease, and cancer are more likely to have coronavirus infection. They are up to five times more likely to die [9]. The COVID-19 mortality rate in Wuhan is reported to be 0.5% for all of the world (4.2%), while the reported mortality rates in Italy, Iran, and Spain are (9.3%), (7.8%), and (0.6%), respectively [10] (Figure 1). [11].

In an open letter to the WHO, 239 scientists from 32 countries wrote about transmitting a new type of coronavirus through tiny particles in the air: The new coronavirus is so small that it can travel through the air and infect people [12].

The data (World Health Organization) show that beginning in March 2020, the pandemic exploded in Europe and the Americas. Since May 2020, the pandemic has increased significantly in America, southeast Asia, and Europe [11].

This study aimed to see how environmental variables affected the COVID-19 virus's survival and behavior, as well as its transmission, in a complex setting. As a result, changes in environmental conditions and human behavior are two drivers of the virus's prevalence. According to the findings, the death rate due to coronary heart disease and the concentration of airborne particles have a statistically direct and significant link. To put it another way, locations with more polluted air have a higher death rate from COVID-19 illness.

## 2. Literature Review

Most individuals can avoid getting sick from COVID-19 thanks to vaccines, but not everyone. Even if a person takes all of the suggested dosages and waits a few weeks for immunity to develop, they still have a possibility of being sick. Because vaccines do not give complete (100%) protection, “breakthrough infections,” in which persons contract the virus despite being completely vaccinated, will happen [13]. In a study by Li et al. [14], a self-recovery humidity sensor was used to monitor wound and respiratory status in real-time. According to Zhuo et al. [15], small molecules targeting mature miRNAs can be discovered through virtual screening strategies. In a feasibility study, Zhuo et al. [16] developed a potentially clinically relevant therapeutic agent targeting MDM2. In Zheng et al. [17], a rule-based reasoning process is proposed based on explicit semantic descriptions of users' unstructured information. The study by Mahmoudi et al. [18] aimed to determine the costs and benefits of COVID-19.

**Figure 2:**
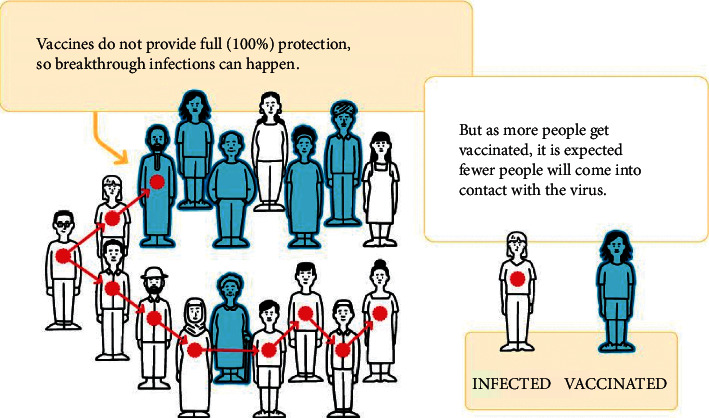
Vaccine protection schematic.

Zhang et al. [19] investigated point-of-care testing technologies for detection of human acute respiratory viruses. Hu et al. [20] developed ultrasensitive point-of-care tests for multiple food safety targets. The study by Chen et al. [21] examined human capital-driven acquisitions. According to Gao et al. [22], a healthy working environment positively impacts corporate innovation, using U.S. Hu et al. [23] reviewed recent publications on COVID-19 and human mobility from the perspective of data-driven analysis of mobility data. Vaccinated persons are more likely to have milder symptoms if they become ill. It is quite unusual for someone who has been vaccinated to have severe disease or die (see Figure 2).

### 2.1. Vaccine Protection and Transmission

COVID-19 vaccinations are essential in the pandemic response because they protect against serious illness and death. Vaccines offer some protection against infection and transmission, at the very least. However, not nearly enough to compensate for the protection they give against catastrophic disease and death. More research is needed to assess how effective they prevent infection and spreading.

**Figure 3:**
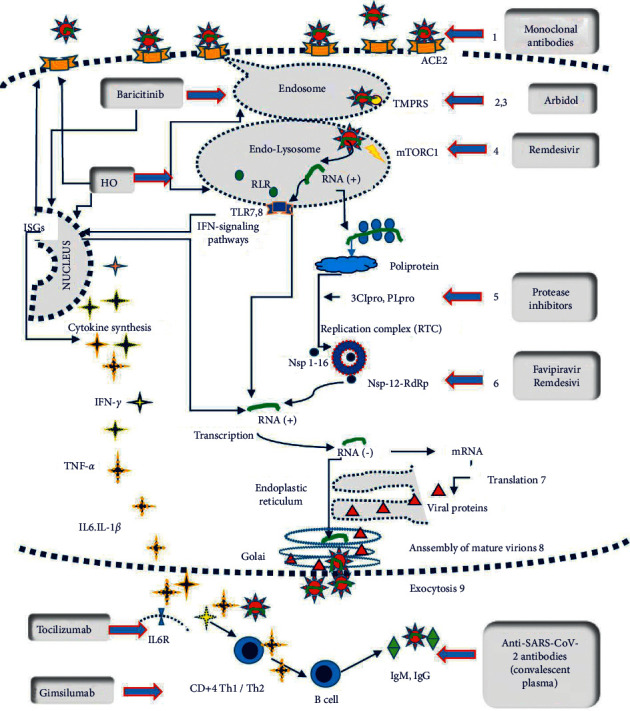
(a) Mechanisms of action of various therapeutic agents being deployed against SARS-CoV-2 and COVID-19; (b) an overview of the various vaccine candidate types and their mechanism of action [24].

After getting vaccinated, people should take simple precautions, such as physical distancing, wearing a mask, keeping rooms well-ventilated, avoiding crowds, handwashing, and coughing into a tissue or elbow. You should still get checked if you are not feeling well, even if you have been vaccinated. Ask your local government for guidance (see Figure 3).

## 3. Virus Transmission Ways

### 3.1. Aerosols and Droplets

**Figure 4:**
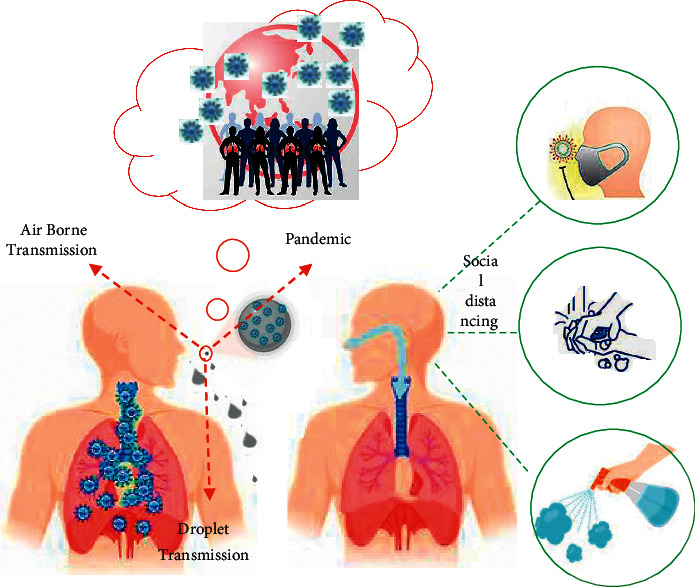
Schematic diagram of aerosol transmission. Most common transmission (red); possible routes of transmission (green).

People with early infection with the virus have no clinical symptoms and can easily transmit the virus [25]. The virus has a very high ability to multiply in the upper respiratory tract [26]. A large amount of the virus has been observed in the swab throat sample of people with and without symptoms [27]. No physical contact is required to transmit COVID-19, which is done by inhaling aerosols in the air [28]. These aerosols are caused by coughing, sneezing, or talking and can stay in the air for hours [29, 30]. Significantly pollute the air and environmental surfaces [31]. The half-life of the virus in aerosols is about one hour [32], which is twice the half-life of influenza viruses [33, 34]. High levels of aerosol transmission of viruses and long-term persistence of aerosols may be the main reasons for the prevalence. The estimated direct infection transmission from each infected person is 1.5 to 6.5 [35]. At the same time, the number of pollution from the environment increases to 5–14 people [10]. Therefore, due to the persistence of the virus in the air, WHO advises people to stay 3 feet (1 meter) to 6 feet away from an infected person [36, 37] (Figure 4). Transportation and the fate of human exhaled droplets play a key role in the transmission of infectious diseases of the respiratory tract. Droplets are sensitive to environmental conditions such as temperature, humidity, and ambient currents. Despite substantial research on SARS-CoV-2, little is known about how the virus spreads via the air; therefore, the researchers utilized mathematical simulations and models to investigate how airflow and fluid flow impact expiratory droplets. Individual particles with density, size, temperature, and humidity have their aerodynamic and thermodynamic characteristics calculated using this simulation model. In typical human breathing, exhaled droplets range in size from a tenth of a micrometer to a thousand micrometers. By comparison, a human hair is about 70 micrometers in diameter, while a typical coronavirus is less than a tenth of a micrometer in diameter. The most common exhalation droplets are about 50 to 100 micrometers in diameter. Drops from an infected person contain virus particles as well as other substances such as water, lipids, proteins, and salts. In this study, researchers examined not only the transport of droplets through the air but also their interaction with the environment, especially through evaporation. The simulations show that in air conditions with 100% relative humidity, larger droplets 100* μ*m in diameter fell to the ground approximately six feet (1.82 m) from the source of the exhalation. But smaller droplets, 50 microns in diameter, can travel longer distances of 16.4 feet (five meters) in humid weather. Less humid air with lower water vapor concentrations can slow this expansion.

This study showed that at 50% relative humidity, none of the 50 *μ*m droplets traveled more than 11.4 feet (3.5 m). The researchers also created a color chart to show how relative humidity and temperature affect the time it takes for a 100-micrometer drop to pass through the air. The diagram showed that when the relative humidity was consistently zero, as the temperature increased, the droplets spent less and less time in the air and then fell to the ground. However, when the relative humidity is about 50%, as the temperature rises, the droplets spend more and more time in the air for up to 12 seconds [38] (see Figure 4).

### 3.2. Skin

**Figure 5:**
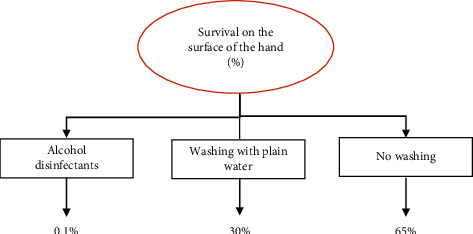
The survival of the 229E (HCoV-229E) coronavirus on the human skin [39]. Survival, expressed in terms of the percentage of viruses remaining on the skin at 37°C. Virus strain tested: human coronavirus 229E (HCoV).

To investigate the survival of the coronavirus in humans, and because of ethical considerations and safety not to permit the use of the COVID-19 virus on the body, the researchers used the human coronavirus, 229E (HCoV-229E) [39]. The results showed that more than 45% of viruses survive on the surface of the hand for an hour, which is much longer than other viruses. The results of another study also showed that rinsing with plain water reduced the HCOV 229E virus by 70%. And if you use alcohol disinfectants, the concentration of this virus will decrease by 99.99% in 30 seconds [40] (see Figure 5).

Skin cells were infected with samples of the coronavirus and influenza A virus by a team from Kyoto Medical University in Japan. The flu virus stayed on the skin for roughly 1.8 hours, according to the findings. The coronavirus, on the other hand, may stay on human skin for up to nine hours, which is four times longer than the flu strain. Information on how long the virus has been on the skin can aid in determining how to prevent transmission through touch and demonstrating the need for handwashing [41].

## 4. COVID-19 in the Environment

COVID-19 disease is now prevalent in most parts of the world [42]. And one of the main concerns is the relationship between the environmental factors and the rapid spread of the coronavirus [43].

**Figure 6:**
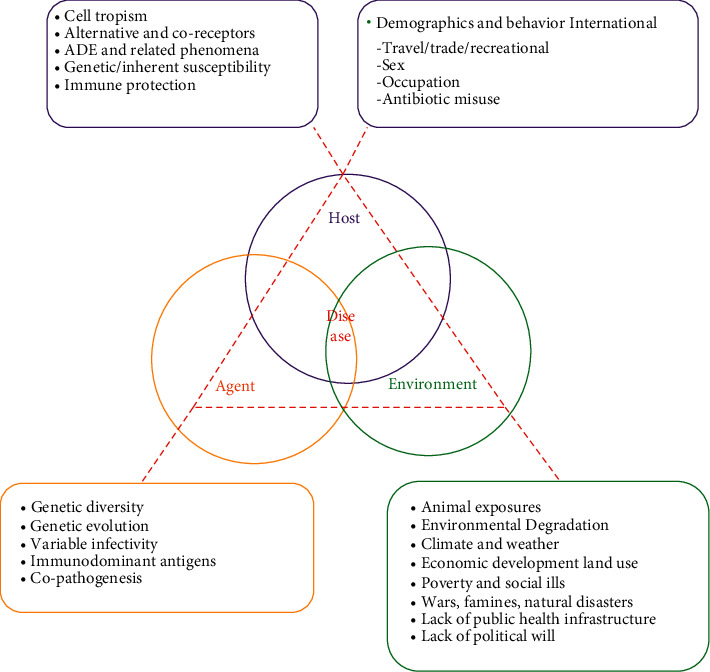
Infectious agents, hosts, and the environment: determinants of disease emergence and persistence diseases [48].

The risk of environmental contamination by SARS, Middle East respiratory syndrome (MERS) virus, and COVID-19 has been reported to be much higher than other viruses [44]. Viruses need harder shells to survive long in hostile environments. COVID-19 has very hard outer layers that protect it from harsh environmental conditions and the human body and increase virus resistance [45]. On the other hand, the long-term survival of the virus outside the body could mean that the virus may need fewer virus particles to transmit the disease [46]. And perhaps this feature also increases its spread. These may explain the high prevalence of COVID-19 and its ability to spread even before the patient begins showing symptoms [47] (see Table 1). Numerous studies have examined the environmental conditions required for the survival and durability of COVID-19, but little is known about the transmission of COVID-19 through environmental factors (see Figure 6).

### 4.1. Atmospheric Particles

Small airborne particles with dimensions of about 2.5 mm are more likely to enter the lower respiratory tract [49–51]. As a stimulant, they cause progressive and chronic inflammation of the immune system [52]. As a result, by the overproduction of mucus, they disrupt the function of the ciliary epithelium and the immune system and predispose people to respiratory diseases and viral infections [53] (see Table 2).

However, several studies have evaluated airborne particles as a damaging factor in epidemic conditions and showed that particulate matter and air pollutants such as ozone (O_3_), sulfur dioxide (SO_2_), and nitrogen dioxide (NO_2_) can stimulate the immune system [55]. Also, people in areas with high levels of air pollution are at greater risk for chronic and infectious respiratory diseases [56]. Based on these results, it is hypothesized that the COVID-19 virus can bind to atmospheric particles. In conditions of air pollution, its durability in the environment increases and is a risk factor for the high incidence of COVID-19 [57, 58]. A study in Italy reported a positive correlation between high levels of air pollution and mortality rate [57], and long-term exposure to air pollutants was significantly associated with the prevalence of the COVID-19 virus. Therefore, it can be concluded that there are still insufficient studies to investigate the relationship between air pollutants and population dynamics to determine the factors that affect the route of transmission of the COVID-19 virus.

Relatively numerous studies have been conducted in India, the United States, Italy, Peru, the United Kingdom, and Germany, showing that short-term and long-term exposure to major sources of air pollution increases the incidence and mortality of COVID-19 [59]. Italian researchers have found that corona has the highest mortality rate in northern Italy, a major industrial part of the country and that air pollution is the highest reported in the region [60]. However, effective environmental factors include vitamin D deficiency, which plays an effective role in the body's defense against the destructive effects of air pollution [61], and air temperature and humidity have played a role in disease and mortality. German researchers also cite low medical facilities and deaths due to corona as having a well-structured medical structure, including well-equipped hospitals, easy access to protective equipment, and the availability of diagnostic tests, as well as low levels of air pollution in the country.

## 5. Climate Indicators

To determine the role of environmental factors on COVID-19, it is necessary to pay attention to the selected period as well as the degree of the concentrated or widespread virus in different regions [62]. COVID-19 researchers focus on epidemiological and climatic data over a wide period to determine the relationship between environmental conditions and transmission rates to maximize similarities. Normalizing this scattered data is also a big challenge [63]. In general, various environmental factors such as temperature [64, 65], pH [66], sunlight [27], humidity [64, 67], and wind flow [68] affect the persistence, transmission, and prevalence of the virus. Various studies have shown that increasing humidity, temperature and wind flow, sunlight, and a sharp change in PH reduce the prevalence of the virus. In examining the relationship between climate and COVID-19 [69], it is important to note that the virus has been able to spread in different climates in different parts of the world [70], and this global distribution has been following latitude and longitude, temperature, and humidity [71]. Therefore, to control the disease, it is very important to confirm the influence of environmental factors on the prevalence of COVID-19. Countries with relatively lower temperatures and lower humidity have a higher prevalence than warmer and humid countries [64]. Alinezhad et al. [72] discuss Bayesian uncertainty analysis in climate change projections. A model of marsh terrace environments in the northern Gulf of Mexico was developed by Osorio et al. [73].

**Figure 7:**
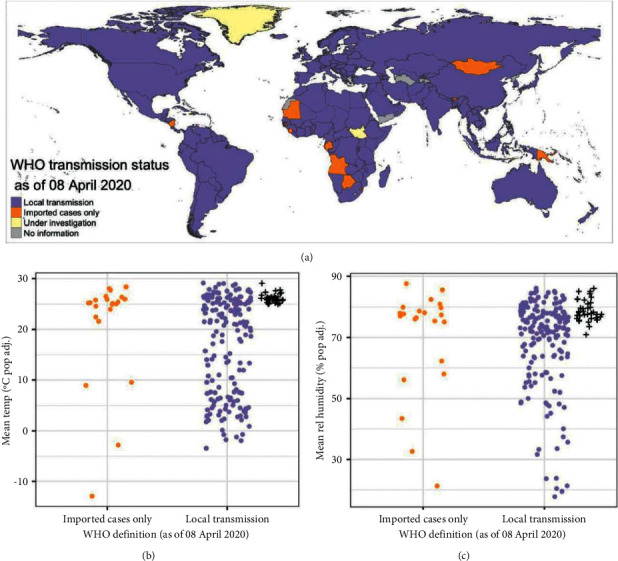
(a) Global map of WHO transmission status for COVID-19 (as of April 8, 2020) and comparison of WHO transmission status with average population adjusted (b) temperature and (c) relative humidity between January 1, 2020, and March 31, 2020.

Belize, Congo, Equatorial Guinea, Fiji, Indonesia, Jamaica, Liberia, Malaysia, Mauritius, Mozambique, Panama, Philippines, Singapore, and Sri Lanka are among the nations having local transmission with an average temperature exceeding 25°C and relative humidity above 70%, which are showed (+) (see Figure 7).

### 5.1. Temperature

The range of temperature changes in the study period has often been small, for example, in the months leading up to the epidemic in China, the temperature range has been between 0 and 20°C [74]. However, only when the temperature is above 30°C does the persistence of the virus in the environment begin to decrease [75]. Therefore, contrary to some researchers, the virus will survive at 30°C, but the survival of the virus is reduced. The resistance of the coronavirus also varies at different temperatures; for example, at 4°C, it can survive for a long time [76] and withstand temperatures of 20°C for up to 9 days. In general, the virus is more active at lower temperatures but dies at 5°C after 5 minutes. Because bats have a body temperature of 48°C, COVID-19 has nothing to do with these creatures [77]. It has also been reported that increasing the temperature by 1 °C reduces the number of new cases by up to 0.86% [78]. A drop in temperature also increased the risk of COVID-19 and mortality by 2.92 times [79].

There is less certainty about the relationship between coronavirus statistics and temperature. Some Chinese research suggests that the number of patients is related to ambient temperature, and some deny this. Researchers say there is no evidence that temperature affects the transmission of the coronavirus and its deaths in Australia, Spain, and Iran. However, high temperatures in Turkey, Mexico, Brazil, and the United States have lowered the incidence, but apparently, a clear threshold has been set [80]. High temperatures do not reduce coronavirus transmission. This has given rise to controversy. Laboratory research shows that the coronavirus is very persistent outside the body at 4°C but loses its persistence at temperatures above 37°C [81].

### 5.2. Latitude and Longitude

**Figure 8:**
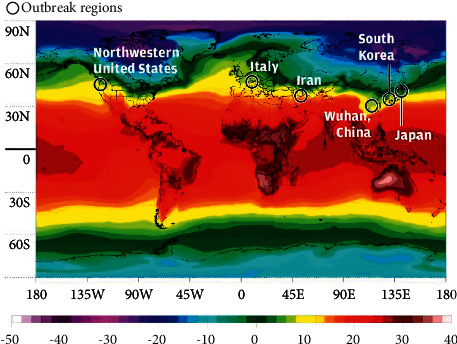
Outbreaks of COVID-19 were located in similar temperature zones [83].

The researchers also found that the most severe outbreaks occur in countries with similar climates, all in a narrow strip 30° and 50° north latitude [82] (see Figure 8). Therefore, the increase in COVID-19 mortality may also be related to the decrease in temperature and humidity in winter [65].

The interaction of temperature and humidity leads us to different patterns of climate, which are determined by latitude. In one study, weather information in 8 cities (Wuhan, China, Tokyo, Japan, Diego, South Korea, Qom, Iran, Milan, Italy, Paris, France, Seattle, USA, Madrid, Spain) with high rates the spread of the coronavirus was compared: these cities were compared with 42 other cities around the world, where the prevalence of the coronavirus was low. The latitude of the first eight cities was between 30° N and 50° N. The temperature of these cities between January and March 2020 was 5–11°C and their absolute humidity was 4–7 g/m^3^. The researchers concluded that “it indicates the behavior of the seasonal respiratory virus.” [84].

### 5.3. Sunlight

A research project in Spain found that the longer the hours of sun exposure after five days of quarantine, the larger the number of persons infected with the coronavirus. When the number of patients and the number of hours of sunshine are separated by 8 to 11 days, this link emerges. There was no link between sunlight hours and patient data before quarantine and during the first five days. This notion contradicts the findings of the influenza study, which demonstrate that the longer the quantity of sunshine, the lesser the disease's transmission. “Another example of a behavioral adaptation that demands less quarantine compliance on bright days is the positive symbol of the sun,” researchers explain. The virus, on the other hand, appears to be unaffected by the sun's UV radiation since the wavelength necessary to kill the virus and bacteria is less than 280 nm. Because it absorbs the ozone layer, this form of ultraviolet (UVC) light does not reach the planet. Human skin and eyes would be badly burnt in minutes if this light reached the Earth. Some small impacts of UVB sunlight, 320–280 nm, have been hypothesized to explain the inconsistent data about the limited transmission of coronavirus in cold and dry circumstances with high latitude. Other variables, like as high vitamin D levels in the bodies of persons living in these places, are, nevertheless, more essential [85].

### 5.4. Humidity

In addition to temperature, the COVID-19 virus is also sensitive to moisture and has a 50% longer lifespan at 30% relative humidity than 30% [86]. Numerous studies have shown that the rate of spread and size of droplets containing the virus through cough depends on the amount of humidity [68, 87]. At humidity above 70%, the droplets are large and have saline physiological conditions that cause viruses to survive [88]. At moderate humidity between 40 and 60%, salt concentrations can inactivate the virus due to evaporation and smaller droplets [89]. At humidity below 30%, the salts crystallize and the virus survives [90]. These results may explain the high transmission capacity of the virus in hot and humid rainy areas. Also in winter with cool and dry air, aerosol droplets are smaller in size and more durable in the air, thus causing the spread of the virus.

The survival of aerosols at 65% relative humidity is about 2.7 hours [51]. In one study, the survival of the human coronavirus 229E (HCV/229E) was investigated under different temperature and relative humidity conditions [91]. The results of this study showed that at 30% and 50% humidity, the virus has a half-life of 27 and 67 hours, while with increasing humidity to 80%, its half-life is reduced to only 3 hours, but if in 80% humidity, the temperature is lowered by 6°, the half-life of the virus is increased by 3 hours and this helps the virus to spread in these conditions. Laboratory and observational studies on patients with coronavirus indicate the effect of moisture on the SARS-CoV-2 virus. Laboratory-produced coronavirus particles had a relatively high shelf life at 53% humidity and 23°C. Even after 16 hours, the virus did not die and was more stable than MERS and SARS-CoV. In this way, it is possible to explain the high rate of its expansion in the form of airborne [92]. Laboratory research does not necessarily predict virus behavior in the real world. However, research on 17 Chinese cities with more than 50 cases shows a link between rising humidity and a decrease in the number of people infected with the coronavirus. One research group measured humidity as absolute humidity or the sum of the amount of water in the air. With each gram of increase in absolute humidity per cubic meter (1 g/m^3^), after 14 days between the increase in humidity and the number of patients, the coronavirus statistic decreased by 67% [78]. Experts report a similar link between the number of cases and humidity in Australia and Spain and the number of cases and deaths in the Middle East. Research on the flu shows that the rate of disease transmission in tropical areas where rainfall leads to moisture is higher in humid and rainy conditions [93]. Researchers say temperatures of 18–21°C and humidity below 11–12 g/kg, roughly equivalent to 13–14 g/m3, are more likely to cause disease in winter. The rate of influenza transmission was higher in tropical countries with higher temperatures and humidity than in the most severe rainfall, more than 150 mm per month [94]. Brazilian researchers have studied the amount of rainfall around the world and confirmed that the incidence of coronavirus increases with increasing rainfall [95]. On average, one inch more rain per day was associated with an increase of 56 new cases of coronavirus per day [96]. The results of a recent study show that the coronavirus, which causes COVID-19, survives 23 times longer in humid air than in dry air. SARS-CoV-2 is carried in microscopic droplets emitted during normal respiratory activities such as breathing and talking [97]. A new study by US researchers shows that droplets can travel up to 16 feet (4.87 meters) in a high humidity environment-high concentrations of water vapor in the air. The researchers claim that high humidity can increase the shelf life of medium-sized droplets in the air by up to 23 times. But dry air with low humidity can accelerate the natural evaporation of droplets and limit the distance they can travel.

### 5.5. Wastewater

The main method of transmitting the COVID-19 virus to humans is through direct contact with the secretions of infected individuals or contaminated surfaces. The virus has also recently been found in feces [98] and urine [99]. Evidence suggests that COVID-19 is less likely to be transmitted through the feces of an infected person, and that gastrointestinal infections and diarrhea occur in approximately 2 to 10% of cases [100]. Several studies have reported the presence of the virus in fecal samples. Although the COVID-19 virus does not withstand temperatures above 26°C [101], it can stay alive on the skin for about 10 minutes [77]. In Oliazadeh et al. [102], algorithms are developed to integrate satellite precipitation data with rain gauge precipitation data to accurately estimate rainfall. The Iranian COVID-19 outbreak was investigated using effective climatology parameters by Ahmadi et al. [103].

**Figure 9:**
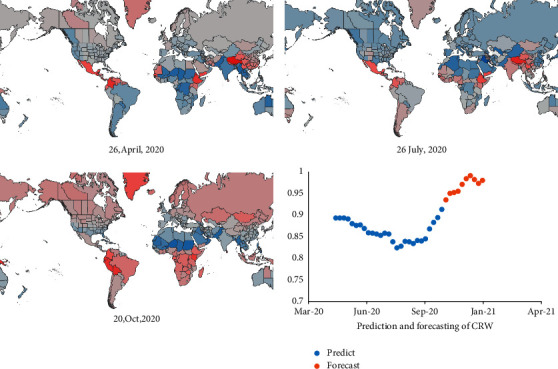
Global relative COVID-19 risk due to weather (crw).

Because the virus is also observed in the feces, the oral-fecal route can be one of the routes of virus transmission. Given that COVID-19 can survive on surfaces for hours or days, therefore, it can be hypothesized that environmental factors such as untreated wastewater, untreated waste, and even soil that can be contaminated with the secretions of an infected person can also transmit the virus. Some studies have reported the existence of COVID-19 virus RNA in untreated wastewater samples [104, 105], but there are no definitive studies on the role of wastewater as a carrier of COVID-19. Some studies have reported the presence of the virus in sewage even when the incidence of the disease was low [106]. According to these reports, the coronavirus can survive for days or weeks in water or sewage sources. Of course, the persistence of the virus in aquatic and wastewater environments depends on other environmental factors such as temperature, sunlight, and organic compounds [107] (see Figure 9). According to the latest WHO report, the human coronavirus has not yet been transmitted through contaminated drinking water [31, 108]. Coated viruses are generally more sensitive to oxidants and may be inactivated faster than nonpathogenic human intestinal viruses in contact with water oxidants [31].

### 5.6. Soil

Limited studies have examined the effect of soil on the COVID-19. Based on previous studies, it is said that the transmission of contamination through soil depends on the soil properties [109, 110]. For example, if the soil is neutral, viruses cannot attach to soil particles. However, the persistence and duration of virus particles in acidic or alkaline soils increase from a few hours to several days. A study has shown that virus-infected areas contain alkaline soils [111].

## 6. COVID-19 on Surfaces

Person-to-person transmission is more likely to occur when infected people have no symptoms or have mild symptoms. COVID-19 can survive on surfaces for 4 to 28 days, but if the temperature reaches 30 to 40°C, the lifespan of the virus is reduced but not eliminated [77]. Studies show that COVID-19 can survive on inanimate smooth surfaces such as metals, glass, and plastics for more than 9 days at room temperature [109], but has a shorter survival rate of 2 days on rough surfaces such as fabric and wood [31]. The most important levels of contamination in centers such as hospitals were mostly related to printers (20%), desktops and keyboards (16.8%), door handles (16%), telephones (12.5%), and medical equipment (12.5%) [112].

**Figure 10:**
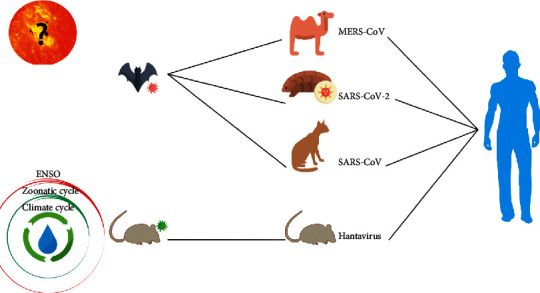
The origin and hosts of different types of viruses (SARS, MERS, COVID-19, and Hantavirus).

Although complete information on the viral load of the surfaces is not available, it can be concluded that by reducing the surface touch and disinfecting the surfaces, the level of viral contamination can be reduced. According to the WHO recommendation, the best way to disinfect surfaces to eradicate the COVID-19 virus is to use ethyl alcohol (62–70%) or hydrogen peroxide (0.5%), or sodium hypochlorite (0.1%) for 1 minute [31, 113]. Several studies on influenza, rhinovirus, coronavirus, and other microbes have shown that infectious diseases, including the new coronavirus, can be transmitted by touching infected surfaces, especially in city centers, offices, and hospitals. Researchers at the University of Massachusetts Dartmouth say touching surfaces such as door handles and elevator buttons are not a major cause of COVID-19 in the United States, but the hypothesis of touching the face is still valid. Infection is transmitted this way: An infected person sneezes or coughs in their hands. Some of the cough or sneeze drops may be sprayed on the surface around the person or the person may transmit the germs by touching a water tube [3]. According to research, the coronavirus lasts up to three days on plastics and steel; but if it sits on a surface, the stable virus usually loses its integrity within a few hours. As a result, a drop that sits on the surface immediately after sneezing is more contagious than a drop that has been sitting on the surface for several days. If you touch infected surfaces and remove enough of the virus and then touch your eyes or mouth or nose, you will get sick if all goes well (see Figure 10).

Other research has used invisible fluorescent detectors, fake microbes that glow under black light, to track the spread of microbes on surfaces. In one set of experiments, 86% of employees became infected with fake germ spray after touching contaminated surfaces. The microbial glow was seen on employees' hands, faces, phones, and hair when spray powder was spilled on the toilet pipe and at the outlet. Fake germs spread from desktop phones to desk, beverage cups, keyboards, pens, and doorknobs. The copier button also contaminated documents and computer equipment. The fake germs landed on backpacks, keys, handbags, house handles, light switches, and kitchen appliances just 20 minutes after leaving home [4]. According to a video posted on the Internet, the black light test has good results. Bright germs landed on the hands of one of the people in the restaurant, and at the end of the meal, everyone behind the person's table became infected. As a result, scientists are banning food from being eaten together during the outbreak of the virus. So far, seven types of coronaviruses have been identified that cause disease in humans [114]. The transmission of COVID-19 from bats to scaly anteaters (pangolin) and then to humans has been reported [115]. The process of transmission between humans is through aerosols. COVID-19 excretion by feces has been reported in some patients [98, 116]. Therefore, mechanical transmission of the virus by insects and arthropods can play an important role in the spread of the disease [31]. Some studies have suggested that household insects, such as beetles, are the main mechanical carriers of pathogens [117] that transmit the disease by contact with contaminated surfaces, patient secretions, and sanitary waste.

## 7. Results and Discussion

COVID-19 is mostly transmitted by aerosols and respiratory droplets, according to several studies. The researchers cautioned, however, that disease transmission by contact with infected surfaces or items should not be neglected, since the coronavirus can live for hours on various surfaces. The findings also back up the theory that good hand cleanliness is crucial in reducing COVID-19 transmission. A hand sanitizer containing 80% alcohol totally inactivates both the coronavirus and the influenza A virus. Handwashing with soap and water or disinfecting with alcohol for at least 20 seconds is now suggested in medical settings [41]. According to studies, particles less than 2.5 microns are the most important air pollutants in densely populated cities. The average concentration of PM 2.5 at the time of the corona outbreak decreased significantly compared to the same period last year in areas of the world where quarantine was carried out completely and not partially during the corona epidemic. But in cases of partial lockdown, the concentration of airborne particles has increased. The reason for the decrease in air quality during this period was due to less use of public transport and increased traffic through private vehicles [118].

Research on the first SARS-CoV in 2003 shows that climate plays an important role in the spread of the coronavirus. Although the virus did not circulate long enough to provide a seasonal pattern, the daily weather was related to the number of cases. The incidence of new cases in Hong Kong's low temperature (below 24.6°C) was 18 times higher than the high temperature. The 2003 epidemic ended in the hot, dry days of July, but strict public health control standards also played a role [119]. New studies on the seasonality of respiratory illnesses explain the effect of cold, dry winter weather on the risk of further transmission of the virus. In this condition, the lining of the nose dries out, which in turn disrupts the function of the villi (tiny hairs in the nasal tube). In this case, the pimple cannot clear the virus from the nose. As a result, the average humidity of 40 to 60% is ideal for respiratory health [31]. Americans spend 87% of their time indoors, so how does the outdoors affect them so much? Humidity is reduced by up to 20% when the cold and dry outside air is combined with warm air inside the room. Indoor humidity is 10–40% in winter but 40–60% in autumn and spring [120]. Low humidity helps to spread the virus's suspended particles and makes the virus more durable. Research shows that the incidence of coronavirus increases with decreasing temperature and humidity. New research points to the severity of the disease in cold, dry climates and which of the following climatic conditions are most associated with the coronavirus. There are good reasons to expect seasonal variation from the respiratory virus. Influenza and respiratory syncytial viruses are more common in balanced regions of the world during the winter. So far, the virus has not shown a seasonal pattern. The obvious problem is that if the pressure is removed from the virus, the virus will resume its life. Oxford researchers in the UK have argued why people should not go to observational research on the number of people infected and confirm the seasonal transmission of the coronavirus to the weather.

Testing capacity has been a serious issue in many nations, according to academics, implying that the number of instances is substantially greater than reported. As a result, any element connected to the weather, as well as a rise in the likelihood of testing, leads us to believe that the number of patients is related to the weather, while the number of tests has raised the number of patients. Other respiratory infections, for example, are widespread throughout the winter months, forcing patients to test for the coronavirus. Milder instances can be found that go unnoticed because they are not accompanied by the symptoms of another respiratory infection. In addition, disorders like cardiovascular disease are more prevalent in cold regions. Patients, who visit the hospital in the winter are more likely to be tested, which increases their chances of being diagnosed. The issue, however, might be caused by other diseases that are impacted by the weather and are not necessarily caused by the coronavirus. People with severe symptoms are expected to go to the hospital regardless of the weather, thus coronavirus fatalities are less likely to be mistaken for testing.

Although the possibility of transmitting the coronavirus through the drinking water and sewage system has been ruled out, credible international articles confirm the possibility of COVID-19 in raw wastewater, but the virus is eliminated in treated wastewater. Although COVID-19 is not resistant to disinfectants and is destroyed, scientists have found that the virus is transmitted through the feces of people with the disease and is present in sewage [121]. It is not possible to transmit the coronavirus through contaminated bodies and soil to groundwater. Because garbage dumps and sanctuaries should not be near drinking water sources and these places should be far from water sources, the depth of groundwater is over 200 meters and at this depth, no microbial agents can penetrate, and almost the possibility of viruses penetrating aquifers. There is no underground. Therefore, drinking water supplied from drinking water aquifers is not a problem [122]. The Centers for Disease Control and Prevention (CDC) has issued a new statement clarifying indirect contact with contaminated surfaces and the risk of developing COVID-19. Get infected with coronavirus by touching an infected surface or object and then touching your mouth, nose, or eyes; But this process is not the main method of spreading the virus [123]. Researchers at Harvard Medical School believe that a long chain of events is necessary to infect people through grocery stores, post offices, outdoor containers, and other surfaces. The final step in the chain is to touch the eyes or nose or mouth with an infected hand; therefore, the best way to ensure chain breakage is to wash the handles [124]. Although similar experiments show the spread of germs on surfaces, germs must survive for a long time and have a large volume to make a person sick. Researchers believe that levels are not effective mediators of virus transmission. For the flu, for example, millions of copies of the flu virus are needed to infect a person by touching the nose; but only a few thousand copies are needed to get infected through the air entering the lungs. Levels are not the main cause of virus transmission, and the CDC is right about that. Surfaces such as door handles and elevator buttons that are touched more often play a more significant role in the spread of viral contamination than surfaces such as food packages [125]. Some research suggests a link between mortality and climate change. The new virus spreads rapidly during a pandemic in a population where no one is immune. “Over the past 250 years, there have been 10 flu pandemics,” the National Academy of Sciences, Engineering, and Medicine said in a statement [126]. The beginning of two pandemics in the Northern Hemisphere was winter, three pandemics in spring, two pandemics in summer, and three pandemics in autumn. “All of these pandemics had a second wave almost six months after the outbreak of the virus in the human population, regardless of when the virus was first introduced.” Using two coronaviruses similar to SARS-CoV-2, which commonly cause colds, researchers developed a model for the prevalence of coronavirus based on weather conditions [127]. The results show that the prevalence of the disease in the community is likely to be so high that it offsets the minor impact of climate change, such as high temperatures and humidity. Using this model, it can be explained why the prevalence statistics in countries that do not comply with health control standards (such as lack of close communication, avoidance of closed spaces, and the use of masks), in hot and humid summer weather ballast.

A team of researchers assessed the severity of the coronavirus rather than the number of patients to overcome the problem of factors other than the weather that distort the presence or absence of coronavirus [128]. With each passing day, the death toll, the average length of stay in the hospital, and the number of intensive care unit admissions decrease at six hospitals in Europe and 13 hospitals in China's Zhejiang Province. In China, the pandemic occurred entirely during winter, while in Europe, the coronavirus outbreak occurred during winter and spring. The death toll in European hospitals dropped with each degree of temperature increase, but not in Chinese hospitals [129]. Researchers ignored treatment advances in February and July and downplayed dexamethasone's effects. [129, 130].

### 7.1. Future Work

After the outbreak of COVID-19 disease in the world of human life, living organisms and their environment were affected in various ways. The spread of the coronavirus has brought many opportunities and challenges to the world environment. With renewable energy, the demand for fossil fuels like coal, oil, and natural gas can be reduced, which results in fewer greenhouse gas emissions. Because of the COVID-19 pandemic, global energy consumption has been reduced, resulting in reduced emissions and improved air quality worldwide. It would be impractical to stop all energy consumption completely in the presence of a pandemic in order to maintain daily needs and global economic development. As a result, using renewable sources of energy such as solar power, wind power, hydropower, geothermal heat, and biomass reduce greenhouse gas emissions while meeting energy needs. The importance of international collaboration in achieving environmental goals and protecting global environmental resources, such as the climate and biodiversity, cannot be overstated. To ensure their proper implementation, responsible international organizations, such as the United Nations Environment Programme (UN Environment), should develop time-bound policies, coordinate international treaties, and coordinate global leaders. To solve the problem and prevent the big problems of post-corona waste by using the experiences of other countries requires an extensive culture of citizens, also city managers must have a permanent place dedicated to each city to have a healthier city.

## 8. Conclusion

Currently, a lack of adequate information about how the virus behaves in environmental conditions has negatively impacted human security and global health. This study aimed to investigate the impact of environmental factors on the survival and behavior of the COVID-19 virus and its transmission in complex environmental conditions. Furthermore, research is needed to determine whether and how the environment could influence the spread of the COVID-19 virus in human society in the long run. COVID-19 is particularly susceptible to extreme temperatures and humidity, and respiratory diseases tend to be affected by seasonal factors, and many of these diseases are at their peak during the winter season. Consequently, changes in environmental parameters and human behavior have been viewed as two important determinants of the prevalence of these viruses. The best way to protect yourself from coronavirus is to get vaccinated, socialize, wash your hands frequently, touch your face, and wear a mask. Ending the COVID-19 pandemic will need equitable access to safe and effective vaccinations. The importance of hand washing extends not only to preventing salivary transmission but also to preventing individual transmission. Droplets of the virus are usually spread by talking, coughing, and sneezing and can be transmitted if one is just one meter away from the infected person. On the other hand, studies show that there is a statistically direct and significant relationship between the rate of death due to coronary heart disease and the concentration of airborne particles. In other words, the death rate from COVID-19 disease is higher in areas with more polluted air. Most likely, the coronavirus will be there at least until the end of next year, and people will have to change their lifestyles to control the disease. It is recommended to pay close attention to hygiene principles such as the use of masks, social distance, and the absence of unnecessary attendance.

## Figures and Tables

**Table 1:** Transmission classification of COVID-19 by country, and territory in the Eastern Mediterranean area [11].

Name	Cases cumulative total	Deaths cumulative total	Transmission classification
*Global*	418650474	5856224	
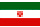 Iran (Islamic Republic of Iran)	6894110	134420	Community transmission
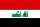 Iraq	2286451	24824	Community transmission
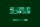 Saudi Arabia	735958	8978	Sporadic cases
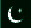 Pakistan	1494293	29917	Clusters of cases
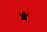 Morocco	1157001	15833	Clusters of cases
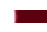 Qatar	352894	616	Community transmission
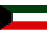 Kuwait	607952	2524	Community transmission
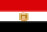 Egypt	461299	23519	Clusters of cases
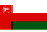 Oman	372060	4225	Community transmission
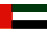 United Arab Emirates	872210	2290	Community transmission
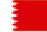 Bahrain	477750	1432	Clusters of cases
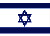 Israel	3515918	9800	Community transmission
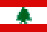 Lebanon	1029998	9890	Community transmission
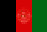 Afghanistan	171673	7524	Clusters of cases
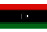 Libya	480945	6169	Community transmission
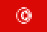 Tunisia	974214	27295	Clusters of cases
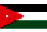 Jordan	1526272	13585	Community transmission
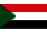 Sudan	59939	3821	Community transmission
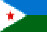 Djibouti	15535	189	Sporadic cases
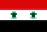 Syrian Arab Republic	53148	3038	Community transmission
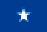 Somalia	26260	1345	Sporadic cases
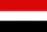 Yemen	11707	2113	Community transmission

**Table 2:** Penetrability according to particle size [54].

Particle size	Penetration degree in the human respiratory system
>11 *µ*m	Passage into nostrils and upper respiratory tract
7–11 *µ*m	Passage into the nasal cavity
4.7–7 *µ*m	Passage into larynx
3.3–4.7 *µ*m	Passage into trachea-bronchial area
2.1–3.3 *µ*m	Secondary bronchial area passage
1.1–2.1 *µ*m	Terminal bronchial area passage
0.65–1.1 *µ*m	Bronchioles penetrability
0.43–0.65 *µ*m	Alveolar penetrability
